# Bio-Inspired Polymeric Structures with Special Wettability and Their Applications: An Overview

**DOI:** 10.3390/polym9120725

**Published:** 2017-12-17

**Authors:** Zihe Pan, Fangqin Cheng, Boxin Zhao

**Affiliations:** 1 Institute of Resources and Environmental Engineering, Shanxi University, 92 Wucheng Road, Xiaodian District, Taiyuan 030006, Shanxi, China; cfangqin@sxu.edu.cn; 2 Shanxi Collaborative Innovation Center of High Value-Added Utilization of Coal-Related Wastes, Taiyuan 030006, Shanxi, China; 3 Department of Chemical Engineering, University of Waterloo, 200 University Avenue West, Waterloo, ON N2L 3G1, Canada; 4 Waterloo Institute for Nanotechnology, University of Waterloo, 200 University Avenue West, Waterloo, ON N2L 3G1, Canada

**Keywords:** bio-inspired, polymeric structures, superhydrophilicity, superhydrophobicity, superoleophobicity

## Abstract

It is not unusual for humans to be inspired by natural phenomena to develop new advanced materials; such materials are called bio-inspired materials. Interest in bio-inspired polymeric superhydrophilic, superhydrophobic, and superoleophobic materials has substantially increased over the last few decades, as has improvement in the related technologies. This review reports the latest developments in bio-inspired polymeric structures with desired wettability that have occurred by mimicking the structures of lotus leaf, rose petals, and the wings and shells of various creatures. The intrinsic role of surface chemistry and structure on delivering superhydrophilicity, superhydrophobicity, and superoleophobicity has been extensively explored. Typical polymers, commonly used structures, and techniques involved in developing bio-inspired surfaces with desired wettability are discussed. Additionally, the latest applications of bio-inspired structures with desired wettability in human activities are also introduced.

## 1. Introduction

**Figure 1:**
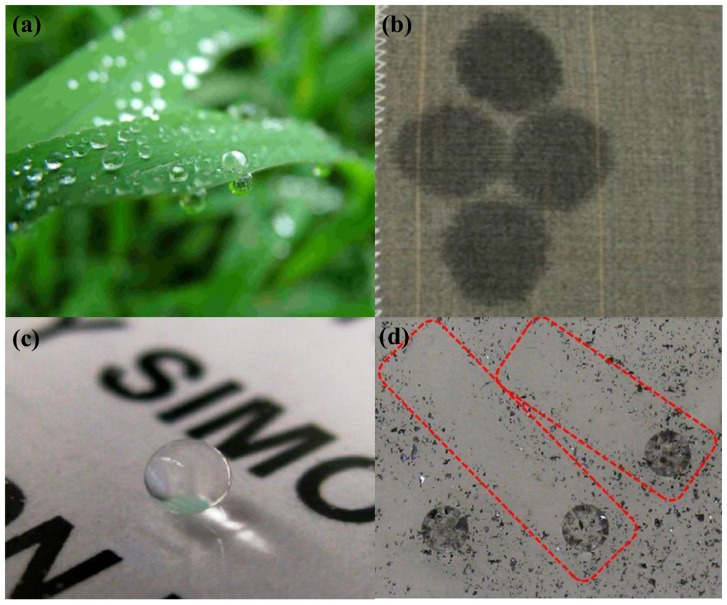
Examples of wetting phenomena in human daily life: (**a**) plant leaf remains non-wetted after rain [1]; reproduced with permission from AIP. (**b**) Water droplets completely wet the fabric surface to leave water stains [5]; reproduced with permission from Wiley. (**c**) Water droplet beads up on transparent superhydrophobic glass slide [6]; reproduced with permission from the American Chemical Society. (**d**) Water droplets roll down carrying away the dusts to leave a clean trace on glass (in red rectangles) [6]; reproduced with permission from the American Chemical Society.

Wetting phenomena can normally be found in nature and are commonly seen in our daily life. Wetting on solid surfaces can be induced either by water (hydrophobicity) or oils (oleophobicity). One classic case of wetting phenomena is defined as superhydrophobicity [1,2,3,4], where extremely high water repellency is found on the surface. Rice leaves illustrate the wetting phenomenon in nature, where water droplets bead up rest on the surface without actually wetting it (Figure 1a) [1]. Another classic wetting phenomena is defined as superhydrophilicity, where water wets and spreads over the surface quickly [1]. Clothes illustrate superhydrophilicity, where fabrics are completely wetted by water, leaving water stains on the surface (Figure 1b) [5]. This is defined as superhydrophilicity [4]. In real applications, superhydrophobic surfaces can be seen everywhere, e.g., water droplets ball up on transparent and superhydrophobic glass slides (Figure 1c) [6] and roll down, carrying away particles and leaving clean traces on glass (red rectangles) (Figure 1d) [6]. For technical applications, wettability is an essential property in painting [7,8], printing [9,10,11], anti-fogging [12,13,14], anti-fouling [15,16,17,18], transportation [19,20,21,22], waterproof products [23,24,25], oil recovery [26,27,28], anti-corrosion [29,30,31], water recycling, etc. [4,32,33,34,35,36,37,38,39] On the micro-/nanoscale, wettability affects micromachining, such as microfluidic channels [40,41,42], nanoprinting [43,44], and lab-on-a-chip systems [45,46].

Inspired by the wetting phenomena in nature, a variety of bio-inspired materials with superhydrophilic/superhydrophobic properties have been developed. An important bio-inspired superhydrophobic structure with high water repellence is inspired by the lotus leaf [32,33,34,35,36,37,38,39], which has been found to play critical roles in nature, human daily life, and industry. It has been well accepted that the hierarchical micro-/nanostructure and the low surface energy are responsible for the superhydrophobicity and excellent self-cleaning properties [2,47]. To date, several classical theories have been devised to comprehensively understand the intrinsic principles behind wetting phenomena, including the Wenzel theory and the Cassie–Baxter theory [2,47]. So far, a series of bio-inspired superhydrophobic structures [32,33,34,35,36,37,38,39] have been developed for self-cleaning [48,49,50,51], anti-biofouling [15,16,17,18,52,53], anti-icing [54,55,56], anti-corrosion [31,57], and adhesion reduction [58,59], etc. Meanwhile, enormous bio-inspired superhydrophilic structures have been fabricated and are similar to bio-inspired superhydrophobic structures but have a relatively high surface energy [1,2,4,14,35,43]. Nevertheless, liquid in real cases (such as oil transportation, oil pumps, and wastewater treatment) usually contains oily components, which easily wet the superhydrophobic surfaces [60,61]. Thus, the fabrication of superhydrophobic surfaces with both water and oil repellency (hydrooleophobicity) will meet the increasing demand of bio-nanotechnology and will largely expand their potential applications.

The design of oleophobic surfaces follows the same principles for fabricating superhydrophobic surfaces, but with a particular emphasis on the reduction of surface energy in combination with surface structures. Fluoropolymers with high amounts of function groups, such as –CF_3_ and –CF_2_ [35,36,37], have the lowest surface energy, thus fluoropolymers have been commonly applied to reduce the surface energy of solid surfaces. Additionally, the trapped air pockets in the patterned structures prevent the penetration of liquid, which contributes to superoleophobicity [36,37]. Thus far, fluoropolymers with modified superoleophobic surfaces (nano-/micro-/hierarchical structure [37,62,63,64,65,66,67,68,69,70,71], micropillars [60,66,72,73,74,75], etc.) have been developed. The typical surface modification approaches in the fabrication of superoleophobic materials are chemical vapor deposition [74,76], electrospinning [70], dip-coating [77,78,79,80,81,82,83], and plasma treatment to graft the fluoropolymer to the surface [71,84,85].

Over the past few decades, a variety of superhydrophobic, superhydrophilic, and superoleophobic structures have been designed to meet performance requirements using copper [86], aluminum [87,88], stainless steel [89,90], silica [91], silicon [92,93], and so on. Nevertheless, metals are difficult for constructing nano-/hierarchical structures since they can easily suffer from defects and corrosion [94]. Inorganic materials are normally lack of sufficient mechanical properties and the reduction of the surface energy is challenging [95,96,97,98]. Therefore, using simple methods to fabricate flexible polymers with desired wettability and properties are regarded as an optimum option. This review mainly focuses on the development of bio-inspired polymeric superhydrophilic, superhydrophobic, and superoleophobic micro-/nanostructures, the techniques involved, and applications. The development of materials with desired wettability and multifunctional properties are also discussed.

## 2. Theoretical Background

**Figure 2:**
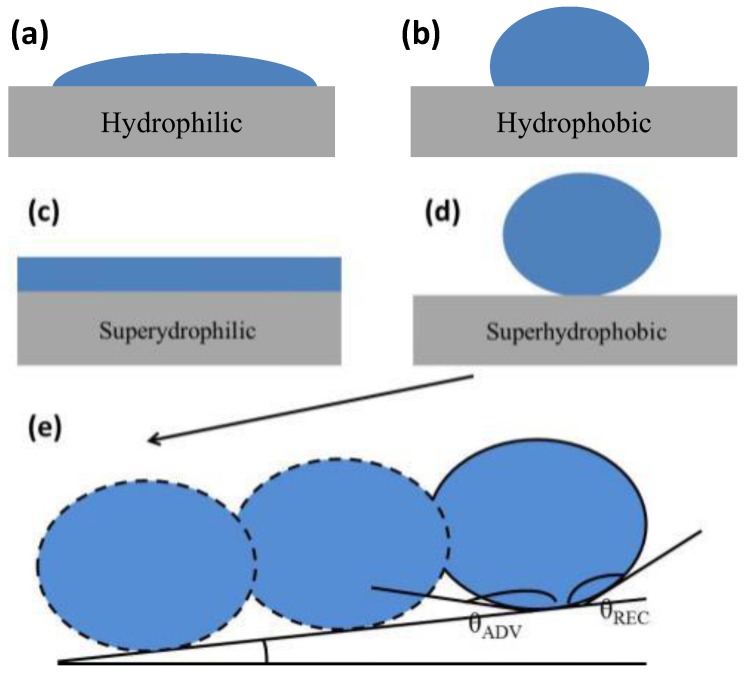
Four general cases of wettability [1]: (**a**) hydrophilic, (**b**) hydrophobic, (**c**) superhydrophilic and (**d**) superhydrophobic; reproduced with permission from AIP. (**e**) Contact angle hysteresis (CAH) *θ*_H_ = *θ*_ADV_ − *θ*_REC_ [39]; reproduced with permission from the Royal Society of Chemistry.

Wetting is the interaction between liquid and solid surfaces [99]; the intermolecular force at the interface determines whether the solid can be wetted by a given liquid or not [1,2,99]. In the process of wetting, the air–solid interface is replaced by an equivalent-sized liquid–solid interface and forms a new liquid–air interface [2]. The system consists of three interfaces in total: a solid–liquid interface, a liquid–air interface, and a solid–air interface. Each interface has specific interfacial energy, and any change in the interfaces causes a variation in the free energy of the whole system [1,2,36]. The change in free energy of the whole system determines the wetting behaviors of the liquid, i.e., whether or not the liquid spreads on the surface [2]. The parameters that most intuitively reflect the wettability of a solid surface are the liquid contact angles, which includes static contact angles and advancing/receding contact angles [100,101,102,103]. Contact angle is defined as the angle between the liquid and solid; its measurement is a common approach to characterize the surface property of the solid surface [102]. Four general cases of wettability have been recognized when water droplets are placed on a solid surface [1]: (a) the superhydrophilic state, where water wets the surface and spreads immediately, leading to a contact angle close to 0° (Figure 2c); (b) the hydrophilic state, where water wets the surface, and the water contact angle is less than 90° (Figure 2a); (c) the hydrophobic state, where the droplet on a solid surface shows a contact angle greater than 90° but less than 150° (120° is the maximum contact angle that water can reach on flat and smooth solid surfaces (Figure 2b)); and (d) the superhydrophobic state, where the water droplet retains a spherical shape on the surface, showing a contact angle usually higher than 150° (Figure 2d) [1].

The contact angle hysteresis is another important parameter for evaluating the interaction between liquid and solid. It depends on surface heterogeneity, roughness, adsorption, deformation, and other factors of the solid surface [1,2,3,4,5,6,7,8,9,10,99,100,101,102]. It is defined as the difference between the advancing and receding contact angles, as shown in Figure 2e [39]. The advancing contact angle is measured at the front of the liquid droplet when it starts to slide, which is the largest contact angle that can be measured on the solid surface [100,101,102]. On the other hand, the receding contact angle is measured at the backside of the liquid droplet when it starts to slide, and it is the lowest theoretical contact angle [100,101,102]. Contact angle hysteresis determines the motion behavior of water on solids [1]. Surfaces with higher contact angle hysteresis require more energy to remove the liquid, because the droplet adheres to the surface. This means more energy is dissipated when liquid flows along such a surface. Surfaces with lower contact angle hysteresis allow a droplet to easily roll off from the solid surface. These aspects are important for designing self-cleaning, fluidic micro-/nano-materials [35,36,37]. 

### 2.1. Young’s Equation

**Figure 3:**
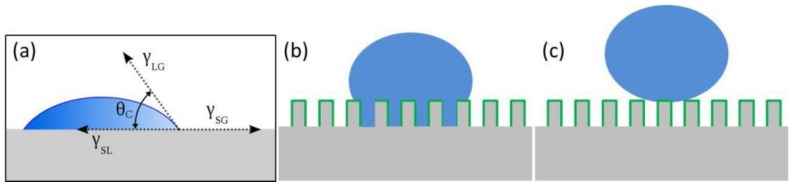
(**a**) Young’s model [34]; reproduced with permission from the American Chemical Society. (**b**) The Wenzel model: a water droplet penetrates into cavities [33]; reproduced with permission from Annual Reviews. (**c**) The Cassie–Baxter model: a water droplet sits on the top of structures [47]; reproduced with permission from the Royal Society of Chemistry.

From a thermodynamics perspective, Young’s equation, as given in Equation (1), provides the basic principle of the wetting phenomenon [35,36,37]. It is established based on the assumption that the solid surface is smooth and flat, and the system is ideal. It neglects influences such as surface roughness, size of droplets, liquid evaporation, surface swelling, vapor condensation, and chemical heterogeneity [36,103]. The contact angle is a balance of solid–gas, liquid–gas, and solid–liquid interfacial forces under thermodynamic equilibrium (Figure 3a) [32,33,34,35,36,39,104].

(1) $$\gamma_{SL}+\;\gamma_{LG}cos\theta_{C}=\;\gamma_{SG}$$

where $\gamma_{SL}$, $\gamma_{LG}$, and $\gamma_{SG}$ are the interfacial tensions of solid–liquid, liquid–gas, and solid–gas interfaces, respectively. *θ_C_* is the equilibrium contact angle. The maximum water contact angle on a smooth and flat surface is approximately 120° due to the large interaction between water molecules and substrate [2]. Roughness (such as microstructure or nanostructure) on such a surface can enhance the water contact angle further (150° < *θ* < 170°) [2]. Thus far, two general models, the Wenzel model and the Cassie–Baxter model, have been proposed to study the effect of surface roughness on wettability.

### 2.2. The Wenzel Model

Wenzel comprehensively studied the effect of surface roughness on wettability, obtaining that the wettability is proportional to the surface roughness of the wetted area [2]. As shown in Figure 3b, in the Wenzel state, water is in contact with the solid surface at all points, including cavities. This results in a greater actual contact area than what is observed (observed contact area calculated from the contact line of water droplet and substrate) [2,99]. The relationship between the apparent contact angle and the surface roughness is described in Equation (2) [2]:

(2) $$\cos\theta^{*}=r\;cos\;\theta_{C}$$

where θ* is the apparent contact angle on the rough surface, and *r*, commonly called the surface roughness, is defined as the ratio of the actual area over the apparent area. Equation (2) indicates that an increased roughness makes a hydrophobic (*θ* > 90°) surface more hydrophobic, while a hydrophilic surface will become more hydrophilic [11]. That is, the surface roughness amplifies the wettability of the smooth surface. However, the wettability of a surface with an extremely high roughness and a porous structure cannot be predicted by the Wenzel model, since an extremely high roughness results in values of cosθ* greater than 1 or less than −1, which is not mathematically possible. In order to solve the problem, Cassie and Baxter developed the Cassie–Baxter model [39]. 

### 2.3. The Cassie–Baxter Model

As illustrated in Figure 3c, in the Cassie–Baxter model, water rests on the protrusions of the structured surface [47]. The trapped air in the cavities is seen as a non-wetting medium, preventing the droplet from penetrating [6,47], so that the droplet can easily roll off when the surface is slightly tilted. The Cassie–Baxter equation relates the apparent contact angle to the contact angle on flat surface:

(3) $$\cos\theta^{*}=\;-1\;+\;\Phi_{S}\;(1\;+\cos\theta_{C})\;$$

where *θ* is the contact angle on an ideal flat surface, $\theta^{*}$ is the apparent contact angle, and *Φ_s_* is the fraction of the solid in contact with the liquid. Compared to the Wenzel model, the Cassie–Baxter model can achieve a $\theta^{*}$ > 90°, even when $\theta_{C}$ < 90° on a flat surface. 

### 2.4. Wetting Transition

In most practical situations, liquid–solid contact might change from the Cassie–Baxter model to the Wenzel model irreversibly due to changes in pressure, evaporation, condensation, or any combination of the three [105,106,107]. The threshold value *θ_Cr_* is defined as the transition point of the Cassie–Wenzel state. Combining the Wenzel and Cassie–Baxter equation, the threshold value *θ_Cr_* can be determined from Equation (4) [36]:

(4) $$\cos\theta_{Cr}=\;\left(\Phi_{S}-1\right)/\left(r-\Phi_{S}\right).$$

**Figure 4:**
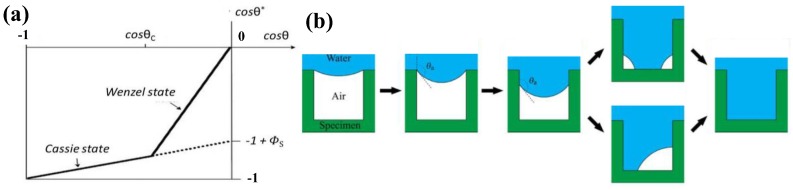
(**a**) The transition between the Cassie–Baxter and Wenzel state [3]; reproduced with permission from the Nature Publishing Group. (**b**) Wetting transition from the Cassie–Baxter state to the Wenzel state [108]; reproduced with permission from the American Chemical Society.

Figure 4a shows the apparent contact angle $cos\;\theta^{*}$ plotted as a function of contact angle cos θ with the solid line displaying the anticipated behavior. The dotted line represents the Cassie regime under a moderate hydrophobicity to stress its metastability [3]. The transition process is complicated, and many factors, such as the Laplace pressure ∆*P* across the liquid–air interface, as given in Equation (5), are involved [36].

(5) $$∆P=\;\frac{2\gamma_{lg}}{R_{d}}$$

where $\gamma_{lg}$ is the surface tension of the liquid, and *R_d_* is the radius of the droplet. 

Two Cassie–Wenzel transition cases have been proposed in the literature (Figure 4b) [105]: touch-down and sliding. The “touch-down” case studies the surface with low pillar height, while the “sliding” case focuses on the case with high pillar height. In the “touch-down” scenario, the droplet penetration depends on the droplet pressure and the critical impalement pressure. This is defined in Equation (6) [105]:

(6) $$P_{imp}=\;\frac{\gamma hR}{CC^{3}}$$

where $P_{\mathrm{imp}}$ is the critical impalement pressure, *γ* is the surface tension of the testing liquid, *h* is the height of the pillars, *R* is the radius of the pillars, and *CC* is the center–center spacing of two pillars. If the external pressure of the water droplet exceeds *P*_imp_, liquid penetrates into the grooves and the Cassie–Baxter model transfers into the Cassie–Wenzel state [109,110,111]. The critical impalement pressure of the “sliding” case is expressed as [105]

(7) $$P_{imp}=\;\frac{2\varphi }{1-\varphi }\left|\cos\left(\theta_{\mathrm{ADV}}\right)\right|\frac{\gamma }{R}$$

where *φ* is the solid fraction, $\theta_{ADV}$ is the advancing contact angle, *γ* is the surface tension of the testing liquid, and *R* is the radius of the pillars. If the contact angle θ is greater than $\theta_{C}$, then the liquid will spontaneously penetrate into the cavities and reach the bottom [97]. Physical features such as the height of the microstructures and the spacing between grooves strongly influence the Cassie–Wenzel transition. C. Ran et al. [107] studied the influence of the diameter and the depth of nanoholes and pointed out that decreasing the diameter and depth of nanoholes can lead to the transition of the Cassie state to the Wenzel state. 

### 2.5. Superoleophobic Surface Design

Due to the extremely low surface tension of oils/organic liquids, it is difficult to achieve superoleophobicity. The principles of developing superoleophobic surfaces are sufficient roughness, trifluoromethyl groups, and special structures (e.g., hierarchical/re-entrant), which follow the roles of designing superhydrophobic surfaces. Besides the theories of Young’s equation, the Wenzel state, the Cassie–Baxter state, and the wetting transition can also be applied to explain the phenomena in superoelophobic fabrication [35,36,37]. 

## 3. Creatures with Special Wettability in Nature

### 3.1. Superhydrophilic Structures

**Figure 5:**
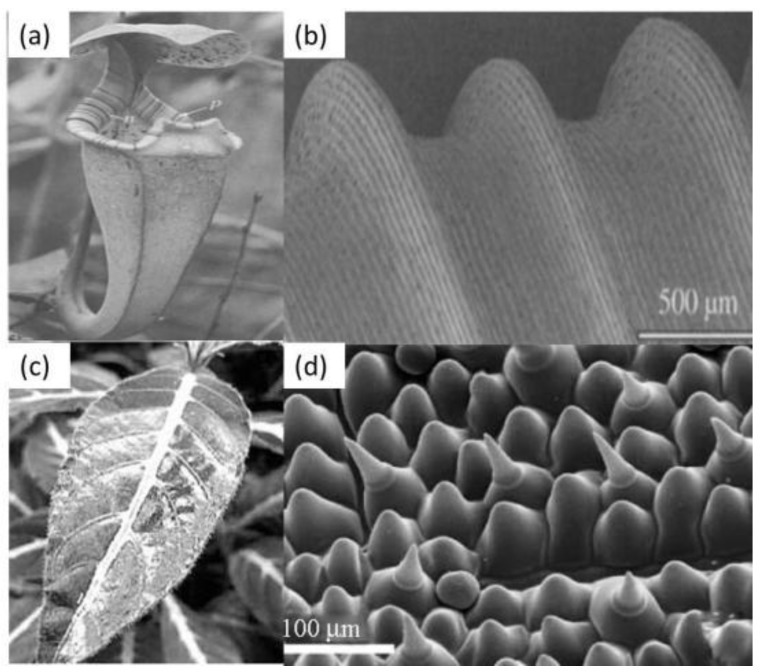
(**a**) Pitcher plant and (**b**) its microstructure [115]; reproduced with permission from The Royal Society. (**c**) Ruelliadevosiana leaf wetted by water with a contact angle of 0° and (**d**) its microstructure taken by SEM [114]; reproduced with permission from the American Chemical Society.

Superhydrophilicity is a characteristic of any surface with a high surface energy. Superhydrophobic surfaces have a water contact angle of less than 10°, sometimes close to 0°, which corresponds to the complete wetting state [112,113,114]. Two important factors for the superhydrophilicity are surface structure and surface chemistry [2,47]. Superhydrophilicity is an important issue for plants in nature, because it involves many activities such as the water and nutrition uptake as well as the activity of photosynthesis [113]. Wetting behaviors on the surface of plants are divided into three cases based on surface structure and chemistry: a smooth surface with high surface energy that is permanently wetted; porous and multiple structure surfaces that absorb water; and hierarchical micro-/nanostructure surfaces for immediate water spreading [1]. In nature, the pitcher plant and the ruelliadevosiana leaf, among others, illustrate the critical role of superhydrophilicity on plant survival. Figure 5a shows the external shape of a pitcher plant which is superhydrophilic [115]. The surface absorbs water moisture to form a thin water film, leading to a slippery surface that can trap insects as food. Its superhydrophilicity comes from two aspects: the covered coating of hydrophilic hygroscopic nectar and its microstructure. The analysis of the microstructure of the peristome revealed that its surface was composed of well-ordered microgrooves with a pitch value of 500 μm [100]. According to the Wenzel theory, surface roughness or structures make a hydrophilic surface more hydrophilic. The microgrooves on the pitcher plant are covered by a hydrophilic hygroscopic nectar, which contributes to the superhydrophilicity. Figure 5c shows 5 μL of water droplets spread on the ruelliadevosiana leaf over 0.2 s (Figure 5c), indicating a superhydrophilic surface [113]. The microstructure of the ruelliadevosiana leaf is different from the pitcher plant, as shown in Figure 5d. The surface has a hierarchical structure containing hair papillae, papillae cells, glands, and channel structures. In addition, surface chemistry analysis of the leaf proved that the gland substrate is hydrophilic. 

### 3.2. Natural Superhydrophobic Structures

#### 3.2.1. Natural Structures with the “Lotus Effect”

**Figure 6:**
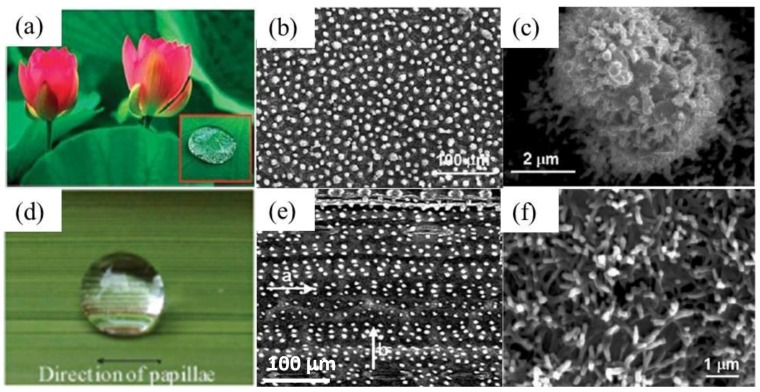
(**a**) A water droplet rests on a lotus leaf without wetting the surface [116]; reproduced with permission from Wiley. (**b**) SEM image of the lotus leaf showing micropapillae on the surface [116]. (**c**) High magnification of a micropapilla consisting of nanonipples [35]; reproduced with permission from The Royal Society of Chemistry. (**d**) A rice leaf with superhydrophobicity [35]. (**e**) The microstructure of the rice leaf, similar to the lotus leaf [116]. (**f**) High magnification of micro-particles of the rice leaf, which are composed of nanoscale fibers [35].

Superhydrophobicity is the ability of a solid surface to repel water, exhibiting a water contact angle ranging from 150° to 180° [47]. The measured water contact angle on a lotus leaf is 162°, (Figure 6a) with a low contact angle hysteresis (CAH) of 2° [116]. The superhydrophobicity and low CAH provides a lotus leaf with a self-cleaning property. Scientists define this property of the lotus leaf as the “lotus effect” [33,34,116]. Microstructure investigation showed that the lotus leaf is composed of micro scale papillae (Figure 6b), and each single micropapilla consists of many nano-sized nipples, forming a hierarchical structure (Figure 6c) [116]. Surface chemistry analysis indicates that the multiple micro-/nanostructure is covered with a thin layer of wax, which reduces the surface energy of the lotus leaf and makes the surface hydrophobic. Additionally, the multiple micro-/nanostructures significantly enhance surface hydrophobicity, so the lotus leaf maintains the superhydrophobic state [2,47,116]. The rice leaf is another common example in nature exhibiting the “lotus effect.” Water droplets bead up on the rice leaf without wetting and spreading (Figure 6d) [35]. However, the water droplet can easily roll off from the surface along the “a” direction, which parallels to the arrays of papillae (Figure 6e). The water droplet adheres to the rice leaf when it is tilted in the “b” direction, which is perpendicular to the arrays of papillae (Figure 6e). The microstructure of the rice leaf is similar to that of the lotus leaf: plenty of well-arranged microparticles are aligned on the surface, and each individual microparticle is composed of nanofibers. The length of these nanofibers reaches 1–2 μm, with a diameter of about 200 nm (Figure 6f) [35]. Also like the lotus leaf, the well-arranged microparticles form two-dimensional structures, displaying varied wettability in each direction. The contact angle in the parallel direction is 153°, and that in the perpendicular direction is 146° [35]. Furthermore, the water droplet can easily slide off along the parallel direction, while the water droplet steadily adheres to the leaf when titled in the direction perpendicular to the arrangement direction.

**Figure 7:**
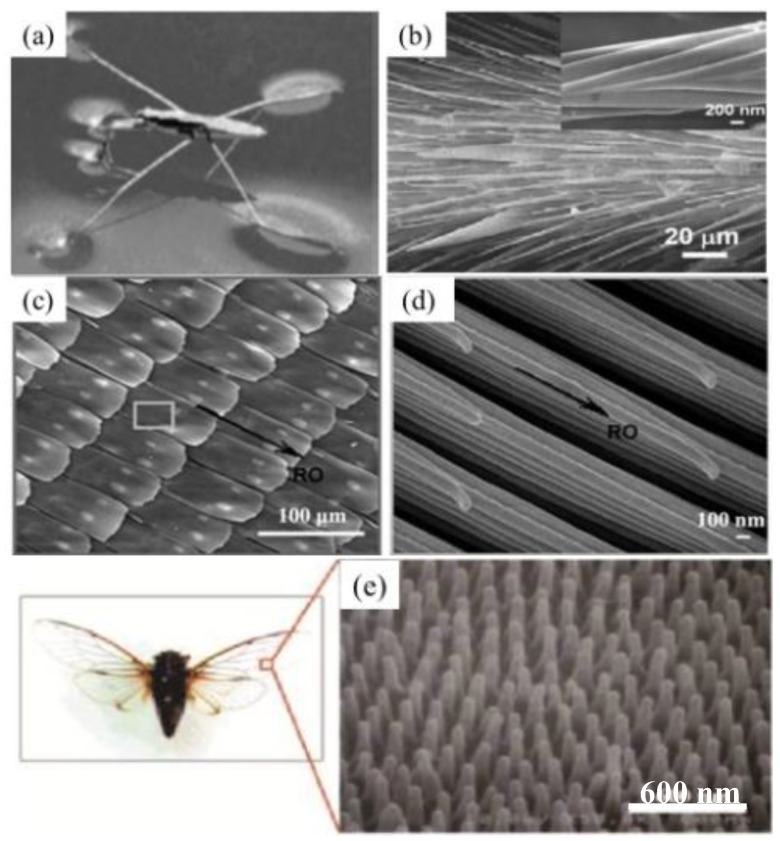
(**a**) Water strider walks on water [117]; reproduced with permission from Nature Publishing Group. (**b**) The micro-/nanostructure of water strider legs [117]. (**c**) The step-like butterfly wing [118] and (**d**) high-resolution microimage [118]; reproduced with permission from The Royal Society of Chemistry. (**e**) The microstructure of cicada wings [121]; reproduced with permission from IOP Science.

Besides plant leaves, which present superhydrophobicity and self-cleaning properties, many insects and animals also exhibit their own specific water repellent and self-cleaning properties as well as drag reduction in fluid flows [117,118,119,120,121]. Several examples with special structures and superhydrophobicity are shown in Figure 7. The water strider can walk quickly on water surfaces (Figure 7a) [117] with its non-wetted legs. Investigation on the microstructure of strider legs has unveiled that water strider legs are covered with a mass of oriented micro-/nano-sized needle-shaped setae (Figure 7b) [117]. These micro-/nano-sized setae structures trap air in the structure, forming non-wetted air pockets that provide a superhydrophobic state with a static water contact angle of 167.6° ± 4.4° [117]. The butterfly is another creature in nature that exhibits superhydrophobic properties. Its superhydrophobic and self-cleaning wings enable it to fly in the rain and stay clean in dirty environments. The SEM image in Figure 7c shows that the superhydrophobicity of butterfly wings is attributed to the hierarchical structures composed of nano-grooves/micro-anisotropic aligned sheets (Figure 7c,d) [118]. Compared to butterfly wings, cicada wings are made of nanoscale pillars. The center–center spacing of nanopillars is around 110–140 nm, and the height of the nanopillars is in the range of 220–250 nm (Figure 7e) [121]. The waxy coating on these regular nanopillar arrays is responsible for the lower surface energy, which explains the superhydrophobicity of cicada wings (a water contact angle of 160°). 

#### 3.2.2. “Petal Effect” 

**Figure 8:**
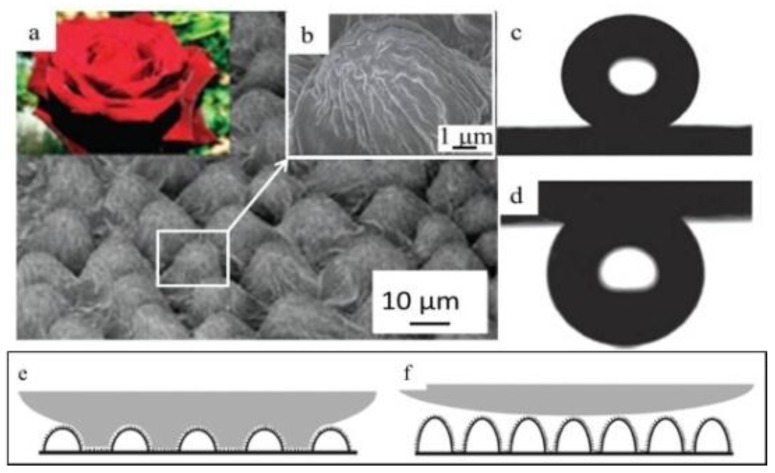
(**a**) The microstructure of rose petals shows that the size of the micropapillae is around 10 μm. (**b**) The high magnification of individual micropapillae shows nano-sized folds [33]; reproduced with permission from the American Chemical Society. (**c**) The static water contact angle on rose petal is 152°. (**d**) The water droplet clings to the surface even when it is placed upside down [33]. (**e**) Water pins to the cavities [122]. (**f**) Water rests on the structures [122]; reproduced with permission from the American Chemical Society.

The other well-known superhydrophobicity phenomenon is the “petal effect” [33]. Surfaces with the “petal effect” show a superhydrophobic state with a static water contact angle greater than 150°, but with a strong adhesion to water [122]. The contact angle hysteresis of the “petal effect” structure is very large. For instance, the water droplet sticks to the rose petal even when it is placed upside down [123,124]. Surfaces with the “petal effect” are not rare in nature, as they are found on garlic cloves, rose petal, lizards, gecko feet, etc. [33,35,123,124]. These materials have broad applications, such as the transport of microliter-sized superparamagnetic liquid droplets by alternating magnetic fields and friction reduction [33,35]. Figure 8a shows the microstructure of rose petals with hierarchical microbumps on rose petals [33]. Many nanofolds were found on the top of a single microbump (Figure 8b) [33]. This multiple hierarchical micro-/nanostructure resulted in a water contact angle of 152° (Figure 8c) [33]. However, the water droplet clings on the surface without moving, even when inverted (Figure 8d), indicating a strong adhesion force [1]. Compared with the lotus leaf, the pitch value (center–center spacing between microbumps) of the microbumps and nanofolds on rose petals are greater, and the depth of the microvallies is much shallower. With these parameters, water can easily penetrate the bottom of the pillar structure, forming Wenzel-state superhydrophobicity, as illustrated in Figure 8e. The water droplet makes full contact with the microstructure, and the left part is held by the structures without penetration, so the surface of the rose petals shows a stronger adhesion to the water droplet. On the contrary, more air pockets are formed between the microstructures with a lower pitch value and higher pillars, as illustrated in Figure 8f. This kind of structure prevents the penetration of liquid and minimizes the contact area between the water and the surface, eventually resulting in a superhydrophobic surface with a lower contact angle hysteresis and a lower adhesion [122,123]. This surface phenomenon that has superhydrophobicity and excellent self-cleaning properties is regarded as a Cassie–Baxter state.

## 4. Bio-Inspired Superhydrophobic, Superhydrophilic, and Superoleophobic Structures and Their Applications

### 4.1. Bio-Inspired Superhydrophilic Structures and Their Applications

Superhydrophilicity is a critical property for living creatures and man-made products. Beetles harvest water using their superhydrophilic shell in desert. Plants ensure their water supply through superhydrophilic leaves. Superhydrophilic surfaces play increasingly important roles in human life: superhydrophilic meshes for oil/water separation [96,125,126,127,128], anti-fogging superhydrophilic glasses [129], superhydrophilic papers for easy printing [130], self-cleaning [131,132], anti-biofouling [133,134,135], corrosion resistance [136], friction reduction [137], etc. As a consequence, a variety of bio-inspired superhydrophilic structures, (i.e., a porous/hierarchical/sponge structure, micro-/nanofibers/pillars/beads/tubes, and polymer coated meshes) [59,96,125,128,129,134,135,138,139,140,141,142] have been developed through the techniques of polymerization [140,143,144], self-assembly [145], deposition [146], immersion [125], electrospinning, etc. [141,147]. Some common bio-inspired superhydrophilic structures, polymers, and modification techniques are summarized in Table 1. 

**Figure 9:**
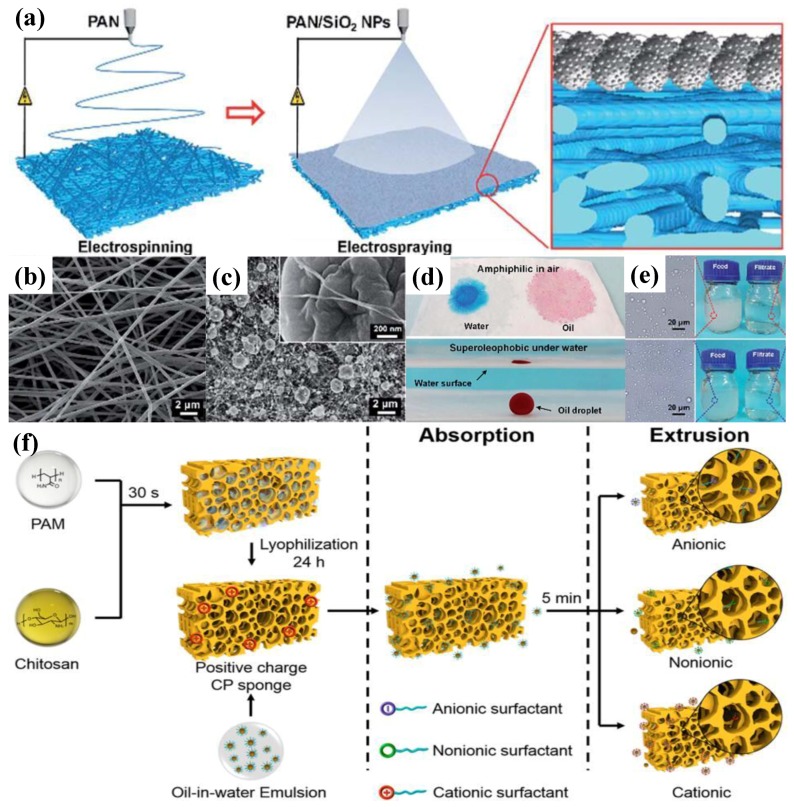
(**a**) Illustration of electrospinning PAN nanofibers/SiO_2_ nanoparticles with hierarchical structures [141], (**b**) a morphology of PAN nanofibers via electrospinning [141], (**c**) SiO_2_ nanoparticle-coated PAN nanofibers with hierarchical structure [141], (**d**) superhydrophilicity and underwater superoleophobicity [141], (**e**) highly efficient oil/water emulsion separation [141], reproduced with permission from the Royal Society of Chemistry. (**f**) Illustration of fabrication superhydrophilic 3D sponge via polymerization [127], reproduced with permission from the American Chemical Society.

Plenty of superhydrophilic structures have been developed over the past few decades. Zhong et al. [144] successfully synthesized superhydrophilicpolypyrrole (PPy) nanowires 60–90 nm in diameter with contact angles of 0° from hexadecyltrimethylammonium bromide (HTAB) and organic diacids via chemical oxidative polymerization. The production turned into nanoparticles 100–300 nm in diameter with the absence of HTAB. Both structures showed a contact angle of 0°, indicating good superhydrophilicity. Ma et al. [148] developed superhydropilic polyvinylidene fluoride (PVDF) through electrospinning assisted by free radical polymerization. The diameter of as-synthesized nanofibers were around 40–100 nm and were wetted by water for 0.2 s. Liang et al. [133] investigated the anti-fouling behaviors of a superhydrophilic PVDF ultrafiltration membrane, which was modified by PAMA-bounded silica nanoparticles via phase separation and post-fabrication. An enhancement in selectivity on Ca^2+^ and Mg^2+^ showed that superhydrophilicity significantly improved the anti-fouling performance of the membrane. The enhanced selectivity was attributed to the secondary membrane, which was formed by the constructed second layer of the silica nanoparticles and the increased porosity after plasma treatment. The changed surface wettability and the hydration layer constructed on the PVDF membrane after superhydrophilic modification endowed the composite membrane with an anti-fouling property. Wang et al. [137] studied the effects of superhydrophilicity on friction reduction using molecular dynamic simulation. Results showed that the properly charged patterns on the polymer surface induced high-ordered water droplets and reduced the friction. Weng et al. [140] developed superhydrophilic zwitterionic polyamide anti-bacterial nanofiltration membranes through interfacial polymerization, and the hydrophilicity was adjusted via the content of *N*-aminoethylpiperazine propane sulfonate (AEPPS) monomer. The as-fabricated membrane showed high water permeability and selectivity. Its anti-bacterial property was enhanced because water molecules were bound to the surface of the membrane, prohibiting the adherence of foulants. Additionally, superhydrophilic materials were reported in detecting gastrodin in aqueous solutions. Ji et al. [142] reported a polymer with excellent superhydrophilicity prepared via molecular imprinting, which showed high selectivity to various organic compounds including gastrodin. The higher gastrodin adsorption capability was attributed to the functionalized superhydrophilicity. Currently, superhydrophilicity is used in water treatment for the elimination of oils and organics. Xue et al. [125] reported a superhydrophilic hierarchical structured mesh for oil/water separation by immersing a stainless steel mesh into a polyacrylamide (PAM) pre-gel solution. The prepared superhydrophilic mesh showed a critically low oil adhesion in water, indicating an excellent self-cleaning property. The separation efficiency of gasoline, diesel, crude oil, vegetable oil, hexadecane, and petroleum ether was greater than 99%. Ge et al. [141] reported that a superhydrophilic hierarchical structured membrane was synthesized by electrospinning polyacrylonitrile (PAN) nanofibrous and then coated by spraying SiO_2_ nanoparticles on the surface (Figure 9a–c). That both water and oil can wet and spread on the membrane verified its superhydrophilicity, and an oil contact angle of 163° under water confirmed its underwater superoleophobicity (Figure 9d,e). Total organic carbon (TOC) measurement indicated that n-hexane–water emulsion was purified with TOC of less than 3 ppm after passing the PAN membrane, indicating a separation efficiency of greater than 99.9%. 

Superhydrophilic membranes have been widely adopted for oil/water separation in wastewater treatment, taking advantage of their high separation efficiency and flux. On the other hand, the separation performances of the superhydrophilic membranes might decline due to their poor mechanical performances and the fact that they are easily contaminated by oils. Thus, new materials that overcome these drawbacks are in high demand. Polymers such as PDMS (polydimethylsiloxane), PU (polyurethane), and polystyrene have shown excellent flexibility and stretchability and are regarded as ideal candidates for superhydrophilic materials [140,141,142,143,144,145,146,147,148]. Xu et al. [127] developed a three-dimensional hierarchical oil/water separation sponge, which produced a transparent milk-like oil/water emulsion over a period of 5 min, by mixing 3% (*w/w*) chitosan with 10% (*w/w*) polyacrylamide (PAM) as a pre-gel solution (Figure 9f). The fabricated sponge had a water contact angle of 0° and quickly spread on the sponge, while the underwater oil contact angle was around 134°, indicating a good oil-absorbing ability.

### 4.2. Bio-Inspired Superhydrophobic Structures and Their Applications

The phenomenon of superhydrophobicity was first noticed on the lotus leaf: water droplets bead up and slide off at a small tilting angle. Later, other superhydrophobic surfaces in nature were found. A hierarchical micro-/nanostructures and a low surface energy are the two dominant factors ensuring a lotus leaf with superhydrophobicity and self-cleaning properties. Nevertheless, the superhydrophobic surfaces in nature are not always self-cleaning. For instance, water droplets strongly adhere to rose petals even when they are placed in reverse because water pins to the cavities adhering to the structure. After the mechanisms behind superhydrophobicity were well understood, mimicking the superhydrphobic structures for practical applications underwent rapid development. A variety of bio-inspired superhydrophobic structures, such as microgrooves [33], nanoparticles [78], micro-/nanopores [81], micro-/nanopillars [14], and micro-/nanofibers [13], have since been developed. The extensive study of bio-inspired structures with superhydrophobicity and remarkable self-cleaning properties has led to the development of bio-mimetic products, such as self-cleaning glasses [6,117], self-cleaning fabrics [149], anti-icing coating [56], erosion prevention surfaces [31,57], and friction and drag reduction coatings [94,150,151]. Many superhydrophobic materials have been reported, most of which have been metals and inorganic materials, but polymeric bio-inspired superhydrophobic structures lack systematic study. Table 2 summarizes the typical structures, polymeric materials, and techniques involved in developing bio-inspired superhydrophobic structures.

**Figure 10:**
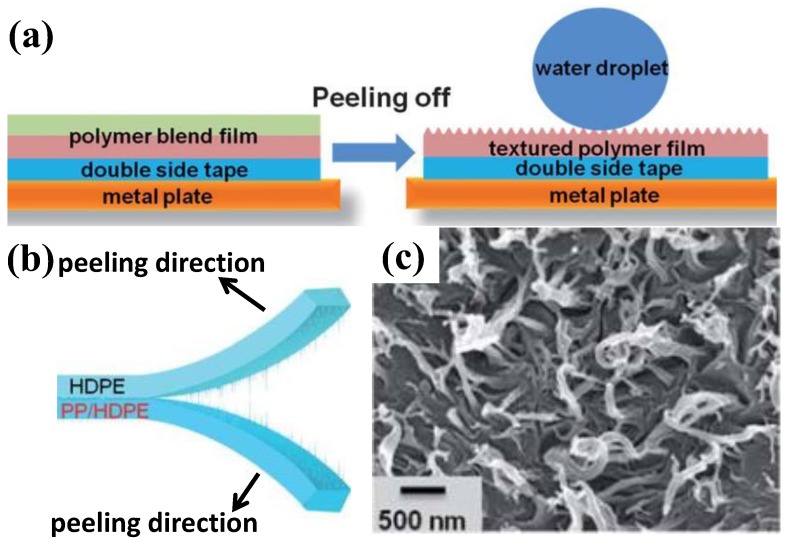
Illustration of using laminating exfoliation to develop a superhydrophobic structure (**a**,**b**) [165] and (**c**) the obtained nanofiber-like structure [165]; reproduced with permission from the Royal Society of Chemistry.

Over the last few decades, a variety of techniques have been reported to develop superhydrophobic surfaces, including electrospinning, spray coating, mold transfer, UV treatment, laser structuring, polymerization, layer–layer assembly, chemical/electro vapor deposition, nano imprinting, dip-coating, and click-chemistry (Thiol-Yne click-chemistry, Huisgen reactions, Staudinger–Vilarrasa reactions) [149,150,151,152,153,154,155,156,157,158,159,160,161,162,163,164,165,166,167,168,169,170,171,172,173,174,175]. Zhang et al. [165] fabricated superhydrophobic and self-cleaning surfaces using laminating exfoliation (Figure 10). In this method, a polypropylene/high-density polyethylene blended film, fabricated via hot compression, and a high-density polyethylene plate were placed on top of each other and then conducted by hot compression of 3 MPa at 200 °C for 2 min (Figure 10a). After the two plates were cooled, they were peeled to separate (Figure 10b), resulting in a nanostructure with superhydrophobicity (Figure 10c). Gong et al. [176] utilized hot embossing to construct a superhydrophobic (water contact angle: 154.6°) and self-cleaning (sliding-off angle: 5.5°) polytetrafluoroethylene (PTFE) surface. Firstly, hierarchical micro-/nano-sized holes on stainless steel were developed assisted by a pico-second laser as a template mold to transfer a hierarchical micro-/nanostructure onto the PTFE. The PTFE was melted and compressed onto the as-prepared mold under a pressure of 55–88 MPa at 150 °C for 2–15 min. The obtained lotus-leaf-like structure was 24 µm in diameter and about 30 µm in height. The nanostructures were around 300 nm, showing a water contact angle of as high as 154.6° and a sliding off angle of as low as 5.5°. Toosi et al. [177] applied femto-second laser irradiation and the template free method to develop superhydrophobic PTFE micro-nano hierarchical structures and investigated the effects of fluence, scanning speed, and beam over-lap on surface morphology and wettability. Results showed that a superhydrophobic surface (160°) was created at the condition of a laser frequency of above 28 J/m^2^ and a scanning speed of lower than 0.5 mm/s. Söz et al. [178] reported a simple spin-coating and cutting approach for fabricating superhydrophobic micro-/nano hierarchical structure on silica/polystyrene and silica/epoxy resin composites, achieving a water contact angle of 163.8°. The polystyrene/silica composites were spin-coated onto glass slides and were cut by a doctor blade (thicknesses of 200, 125 and 50 mm), obtaining a hierarchical superhydrophobic structure. Liu et al. [50] explored the approach of spray-coating PMMA/PDMS onto a substrate and achieved a superhydrophobic (water contact angle: 164.3°) and self-cleaning (sliding-off angle: 3.2°) porous surface. 

Superhydrophobic structures have broad applications in practical activities, such as oil/water separation, anti-icing, ice adhesion reduction, self-cleaning surface, wear-resistance, friction reduction, thermal stable, transparent, drug release, antibacterial, and adhesion reduction [149,150,151,152,153,154,155,156,157,158,159,160,161,162,163,164,165,166,167,168,169,170,171,172]. Currently, the widespread applications of superhydrophobic materials are oil/water separation [179], anti-icing [180,181], and anti-contamination [182]. Water pollution caused by oily species threatens human health and has triggered serious environmental problems. Ice accumulated on exposed surfaces (power lines, air craft, offshore oil platforms, and wind turbines) causes equipment failure and/or serious accidents, resulting in great economic loss [56,180,183,184,185,186,187]. Surfaces (such as those of a ship or engine) contaminated with dust, bacteria, and other waste can cause an increase in drag force, corrosion, and the loss of function [31,48,50,57,97,151,153,154,155,188,189,190,191,192,193]. 

**Figure 11:**
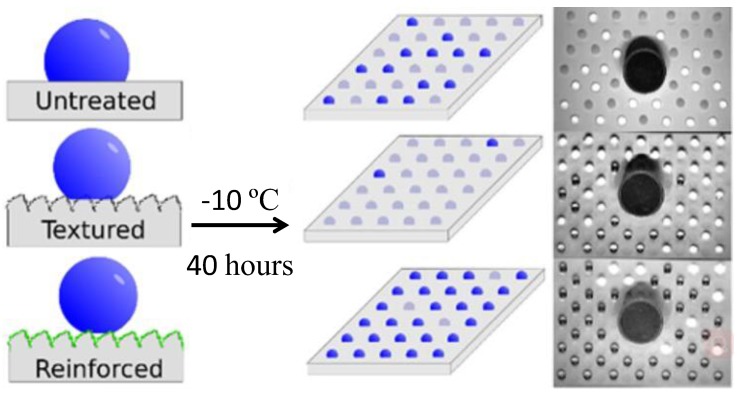
Reinforced superhydrophobic coating on silicone rubber for excellent anti-icing performance under −10 °C [195]; reproduced with permission from the American Chemical Society.

A superhydrophobic and superoleophilic polyester–PDMS porous membrane was developed by spray-coating for oil/water separation [194]. The obtained membrane had a diameter around 0.1–0.8 µm and showed excellent superhydrophobicity and self-cleaning properties, with a water contact angle of 163° and a sliding off angle of 2.3°. By increasing the concentration of the copolymer solution, continuous pores with a diameter ranging from 40 to 150 nm were formed. The oil/water separation efficiency of these fabricated membranes was higher than 99% and the membranes were reusable after oil/water separation. Another important application of superhydrophobic materials is anti-icing on surfaces. Superhydrophobic micro-/nanostructures have been shown to be effective in delaying freezing due to the slight contact between the water and the substrate. Furthermore, it is well known that surfaces with higher water contact angles can reduce the formation rate of ice nuclei, which is what anti-icing consists in [195,196]. Emelyanenko et al. [195] reported that superhydrophobicity (water contact angle: 168°) and excellent self-cleaning properties (sliding-off angle: 2.5°) were achieved through the laser texture method and through fluorooxysilan-coating using PDMS and silica nanoparticles as composites (Figure 11). The anti-icing experiment carried out under −10 °C and −18 °C showed that water droplets retained a metastable state on hydrophobic and superhydrophobic surfaces in a super-cooled atmosphere, and the work of adhesion increased with the decrease in the water contact angle, indicating improved the anti-icing properties of the superhydrophobic structure. 

Though extensively artificial superhydrophobic structures have been developed, surfaces contaminated by bacteria, oils, dust, and other waste adversely affects their performances. Fu et al. [51] studied the anti-bacterial, superhydrophobic, and self-cleaning surfaces which were brought about by the durable self-cleaning performances and dominates the long-life time application of superhydrophobic materials. In their study, poly(urea-formal-dehyde) (PUF) and hexamethylenediisocyanate (HMDI) were used to construct superhydrophobic structures via copolymerization, spray-coating, and thermal curing. The surface morphology and wettability were tuned via the content of PUF and HMDI. The largest water contact angle was 162.3°, and the lowest sliding-off angle was 3.2°. This outstanding superhydrophobicity and relatively small rolling-off angle enabled water to carry away contaminates. Furthermore, the superhydrophobic surface was functionalized with quaternary ammonium salts, which adhered strongly to negative bacteria such as *S. aureus*. This functionalized property, combined with superhydrohobicity and self-cleaning, contributed to its anti-bacterial performance. 

**Figure 12:**
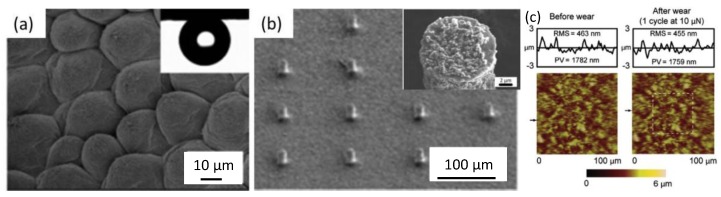
(**a**) Bio-inspired rose petal structure with strong adhesion [124]; reproduced with permission from the American Chemical Society. (**b**) Hierarchical micro-/nano-pillar showing superhydrophobicity and strong adhesion [123]. (**c**) Wear test performed by AFM [123]; reproduced with permission from Elsevier.

**Figure 13:**
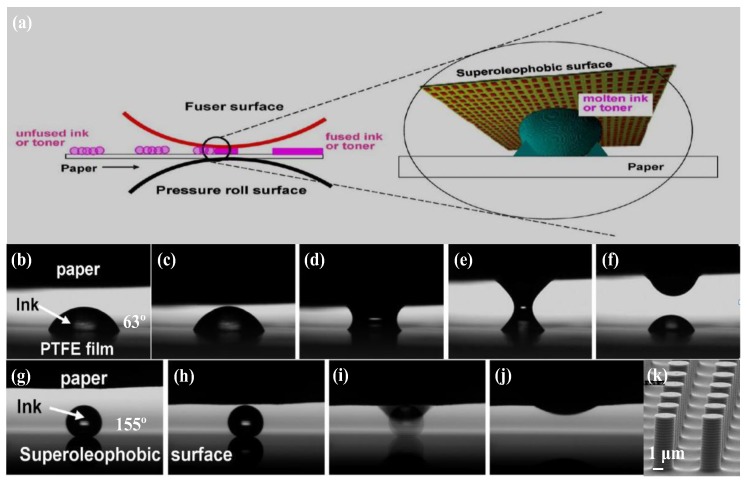
(**a**) Basic principle of the fuser with the superhydrooleophobic surface. (**b**–**f**) Illustration of the interaction between paper and molten ink droplets on an oleophilic PTFE substrate during the printing process. Part of the ink was left behind on the PTFE film and not completely printed onto the paper (**f**). (**g**–**j**) Illustration of the interaction between paper and molten ink droplets on superoleophobically textured silicon pillars. Ink was completely printed onto the paper (**j**). (**k**) shows the textured superoleophobic pillar arrays used in the printing process [73]; reproduced with permission from the American Chemical Society.

Feng et al. [124] mimicked a rose petal structure using polystyrene, and the duplicated structure is shown in Figure 12a. Water droplets stuck to the surface even when they were placed upside down, implying superhydrophobicity and strong adhesion. Ebert et al. [123] studied the wear resistance of the bio-mimetic rose petal structure via a replicating microstructure of a Si micromodel. The overall structure consisted of low-density micropillars with large center–center spacing, as shown in Figure 12b [123]; the inset image shows the single pillar’s surface, which was covered by nanoparticles. This hierarchical structure gave rise to strong adhesion to water, not dropping from the surface when it was inverted. The evaluation of roughness performed under AFM (Figure 13c) indicated that the surface was anti-wear (Figure 12c). The root mean square roughness (RMS) and peak–valley roughness (PV), which were 463 and 1782 nm before the test, respectively, were 455 and 1759 nm after sliding, respectively.

### 4.3. Bio-Inspired Superoleophobic Structures and Their Applications

#### Bio-Inspired Superoleophobic Structures

Generally, materials with only superhydrophobicity can be contaminated by organic oily species [60], which have surface tensions much lower than water. Materials that are both superhydrophobic and oleophobic are in high demand for many applications [150], such as anti-biofouling [182,197,198] for ships, non-stick [73] and corrosion-resistant [68] protective coating, cleaning polluted water [199], and anti-bacterial [200] coatings for cotton fabrics and microfluidics [53,201] in lab-on-a-chip systems. To fabricate materials that are able to achieve superhydrooleophobicity, bio-inspired micro-/nano- patterned structures [14,15,20] combined with surface energy reduction treatment [60] have been developed. Among those reports, fluorine compounds with a high percentage of –CF_3_ and –CF_2_ groups have been applied to reduce the surface tension of the patterned surface [197,198,199,200,201]. Table 3 shows examples of recent developments in the fabrication of bio-inspired oleophobic structures. Several surface modification approaches such as vapor deposition, liquid deposition, and electrospinning have been used to fabricate the biomimetic oleophobic materials [62,63,64,65,66,67,71,75,87,150,191,202,203,204]. Even then, however, plasma treatment on superhydrooleophobic polymer surfaces may cause surface damage (such as cracks) [150], and the coating approach can worsen the surface roughness and cause surface defects, ultimately reducing the oleophobicity [205,206]. 

The comprehensive understanding of surface roughness/structure and surface energy on superhydrophobic structures inspired the development of superhydrooleophobic structures. The topics relating to oil adhesion, including bio-adhesion, microprinting, self-cleaning in oil-polluted water, antifouling, oil adhesion reduction, oil transportation and oil/water separation, have attracted significant attention [197,198,199,200,201,202,203,204,205,206,207]. On images from a printer, for example, the melted ink is supposed to print onto paper and adhere completely without adhering to the fuser roll. This requires the fuser roll surface to have a self-cleaning property, which can be achieved by functionalizing the surface with superoleophobicity. Zhao et al. [73] designed a superhydrooleophobic structure applied in paper printers, aiming to reduce the high adhesion between the fuser surface and the paper during the fusing process. As illustrated in Figure 13a, the melted toner or ink separated from the superoleophobic fuser surface without any residues, thus improving the efficiency of printing [73]. The adhesion behavior of melted ink was compared on an oleophilic surface (oil contact angle of 63°, Figure 13b–f) and a superoleophobic textured micropillars surface (oil contact angle of 155°, Figure 13g–k). When the ink was melted and detached from the fuser surface (Figure 13b–f), there were ink residues left behind due to the oleophobicity and a strong adhesion to the fuser surface. However, the melted ink separated from the fuser surface completely without remaining residues (Figure 13g–j), indicating that the superoleophobic surface had low adhesion and excellent self-cleaning properties.

In addition, the superoleophobic materials play much more important roles in water/oil separation and oil transportation. In the water/oil separation industry, permeable membranes are commonly used materials [208]. However, most of these membranes are oleophilic with non-negligible oil adhesion, which lead to severe oil contamination [96]. Preventing the oil from penetrating the membrane can further decrease the water/oil separation efficiency. As refer to oil transportation, oil can easily form hydrates and these hydrates adhere to the inner walls of pipelines generating a dramatic increase in drag force, leading to a greater energy requirement for transporting the oil.

**Figure 14:**
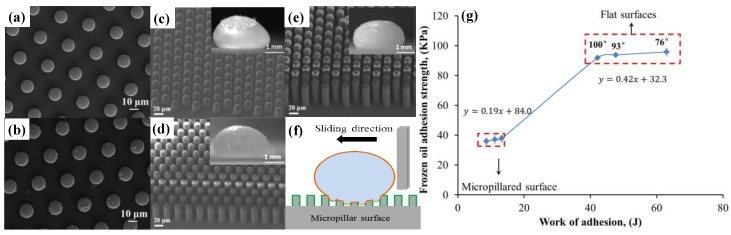
(**a**,**b**) The overall structure of trichloro(1*H*,1*H*,2*H*,2*H*-perfluorooctyl)silane (FDTS) blended and coated PDMS micropillars, respectively [216]; reproduced with permission from Elsevier. (**c**–**e**) The micropillars with aspect ratios of 1.5, 3.0 and 4.5 and the corresponding frozen oil droplets [217]. (**f**) The carton illustration of frozen oil knock-off test [217]. (**g**) The systematic relationship between frozen oil adhesion, surface structure, and surface composition [217]; reproduced with permission from the American Chemical Society.

Oil adhesion has attracted extensive studies over the past few decades due to its important role in many industrial applications such as lubrication, lithographic etching, and anti-contamination [63,204,209,210]. Ambient temperature behaviors on varied surfaces ranging from oleophilic to superoleophobic have been well investigated. However, there is insufficient understanding of oil adhesion behavior at low temperature, especially when the oil is frozen at sub-zero (Celsius) temperatures. Such low-temperature phenomena are very important in scenarios such as oil freezing in engines or in oil transport pipelines, where the freezing leads to a dramatic increase in adhesive force, which requires substantial amounts of energy to overcome, potentially causing instrument failure as well [211,212,213,214,215]. Pan et al. [216,217] extensively studied the relationship between oleophobicity and frozen oil adhesion reduction by fabricating flat surfaces and superoleophobic micropillars with different aspect ratio (Figure 14). These superoleophobic micropillars were constructed by blending fluoropolymer into PDMS via mold transfer. This blending approach showed its advantages on maintaining durable and better oil repellency comparing with traditional vapor deposition method (Figure 14a,b). The shapes of frozen oil droplets (kept at −30 °C for a while) were recorded, and the frozen adhesion forces were measured via knocking-off the frozen oil droplets (Figure 14c–f). The work of adhesion calculated and plotted against the frozen oil adhesion strength indicated a notable reduction in adhesion strength from superoleophobic micropillars (Figure 14g). 

The literature on the adhesion between frozen oil and micropatterned surfaces is limited; but many efforts have been devoted to exploring possible strategies to remove ice from surfaces, which can all be categorized into either passive strategies or active strategies [184,185,218]. The passive strategy is implemented either by controlling the surface wettability to reduce ice adhesion or by applying mechanical forces to remove the accumulated ice [184,185,218]. This is because superhydrophobic surfaces can be used to delay the ice formation and reduce the contact area, thereby reducing the shear forces between the ice and substrates. However, such tests are only conducted in laboratory research; the practical application of using superhydrophobic coatings alone is not an ideal method. Mechanical elimination is difficult to conduct on a large scale in actual cases. The active strategy employs external energy such as thermal, chemical, or pneumatic to eliminate the accumulated ice [183,219,220]. Among these methods, thermal melting uses sources such as warm air or electrical current and is regarded as one of the most effective ways to remove ice [183,219,220]. One decade-old traditional approach is warm air melting, in which intense warm air is blown onto objects through a special tube [184,185,218]. This raises the temperature of the system quickly, resulting in the melting of ice [183]. However, this requires a high consumption of energy at low temperatures and is more difficult to operate in an environment with strong winds at present. Currently, electrical joule heating is widely utilized due to its lower energy cost and easy operation [186,187,221]. Superhydrooleophobic materials show excellent water/oil repellence; so they can prevent the surface from being contaminated by oil, bacteria, or dust. Generally, superhydrooleophobic materials have been well studied and applied in the areas of self-cleaning, anti-corrosion, and antifouling. However, most superhydrooleophobic materials are not electrically conductive, which restricts their applications in the fields of conductive materials such as skin sensors, electrically controlled or signal collecting systems, radiofrequency antennas, and lab-on-a-chip systems [222,223,224,225,226,227,228], which play important roles in everyday life and industry activities.

**Figure 15:**
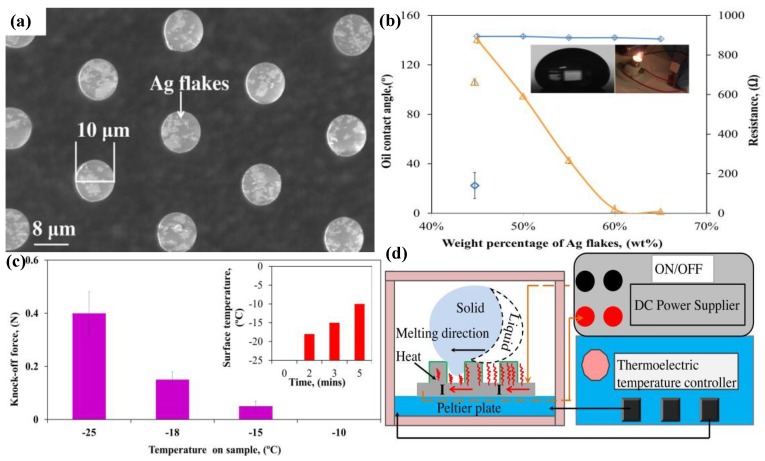
(**a**) SEM image of Ag flakes/epoxy resin composite micropillars. (**b**) Effects of Ag content on superoleophobicity and electrical resistance. (**c**) Relationship between knock-off force and surface temperature. (**d**) The Joule heating effect [229]. Reproduced with permission from Elsevier.

Although the fabrication of electrically conductive polymer composites have achieved significant success during the past years, blending conductive fillers into a polymer monomer, electrospinning conductive polymer (polypyrrole) nanofibers, and embedding Ag nanowires into polymer surfaces [222,223,224,225,226,227,228]. Nevertheless, they were not water- or oil-repellent. As a consequence, it is important to develop superhydrooleophobic materials with electrical conductivity to solve the problem of frozen oil adhesion. Carbon nanofiber (CNF)-reinforced fluoroacrylic co-polymer nanocomposites were fabricated by Das et al. [67] The oleophobicity of the nanocomposites changed from oleophobic at a concentration of 10 wt % (CNFs) to superoleophobic at 60 wt %. The oil contact angle hysteresis (9°) was small at high CNF concentrations (higher than 40 wt %), while the nanocomposite surfaces showed strong adhesion to oils at low concentrations of CNFs (10 wt % and 20 wt %). The low oil contact angle hysteresis indicated that the surface had remarkable self-cleaning properties. The conductivity of the nanocomposites reached as high as 1 × 10^3^ S/m at a concentration of 60 wt %. Pan et al. [229] fabricated superoleophobic conductive micropillars to reduce frozen oil adhesion by blending Ag flakes into an epoxy resin and then utilized the mold transfer approach. FDTS was covalent bonded to the surface of micropillars during mold transfer to delivery superoleophobicity. The fabricated composite micropillars were 10 μm in diameter, 68 μm in height, and 25 μm in center–center spacing (Figure 15a). The study found that an increasing content of Ag flakes in epoxy micropillars had negligible effects on the oleophobicity, while the electrical conductive resistance decreased dramatically with the increase in Ag flakes in epoxy (Figure 15b). The electrical resistance dropped from about 800 ohm to several ohm. Significant Joule heating can be generated at relatively high electrical resistance, resulting in an increase in the surface temperature of the micropillars. Frozen oil adhesion decreased with increasing surface temperature (Figure 15c). Frozen oil adhesion reduction was carried out through the electrical-current induced Joule heating and hot wind melting. Frozen oil adhesion could still be measured at 0.05 N at a temperature of −5 °C by hot wind melting, while frozen oil adhesion was 0 when the surface was heated up to −10 °C by the electrical-current induced Joule heating. Surface temperature was dominated by resistance and the generated Joule heating. Larger resistance produced more heat and thereby a higher surface temperature. This difference may contribute to the different melting processes of the frozen oil droplets. When a DC current is applied, the frozen oil starts to melt from the bottom and transfers the solid–solid contact into a solid–liquid contact, reducing the adhesion force, while the hot wind melting started to melt from the top and the solid–solid contact did not change until the bottom of the frozen oil droplet was melted, causing greater adhesion force (Figure 15d). 

## 5. Conclusions and Perspective

This review article summarized the recent development of bio-inspired superhydrophilic, superhydrophobic, and superoleophobic structures inspired by plant leaves (e.g., the lotus leaf and the rose petal) and creatures (e.g., butterfly wings, water strider legs, and shark skin) that have employed a variety of polymers and techniques. By reviewing the theory of wettability, a series of studies that investigate the effects of surface energy (low/high surface energy) and surface structures (nano-/micro-structure, hierarchical structure) on delivering desired wettability has been launched. The techniques used for developing surfaces with desired wettability are similar, but the requirements for surface energy to delivery superhydrophilicity, superhydrophobicity and superoleophobicity are different. Oil has a much lower surface tension and can easily wet superhydrophobic surfaces. Superhydrophilic surfaces require an extremely high surface energy and a proper structure, while superhydrophobic and superoleophobic surfaces require a low surface energy. Repelling oils for superoleophobic surfaces especially requires a critically low surface energy. Bio-inspired surfaces with special wettability are applied widely in oil/water separation, anti-wear, anti-fouling, self-cleaning, friction reduction, drug release, adhesion reduction, etc. With the rapid progress in technology, bio-inspired super-wettable surfaces with advanced properties are in high demand. 

However, the current development on constructing bio-inspired super-wettable materials has disadvantages. Most polymeric bio-inspired materials lack sufficient mechanical properties and advanced multifunctional properties; contaminates on surfaces cause a loss of function and lifetime; the ease of damage and defects in current construction techniques as well as short lifetimes increase its production costs. In the near future, it is crucial to develop bio-inspired super-wettable materials with high mechanical properties and multifunctional performances with simple techniques to enhance their lifetime and durable wettability.

## Figures and Tables

**Table 1:** List of bio-inspired structures, materials, and surface modification techniques for the fabrication of bio-mimicked superhydrophilic structures.

Structure	Materials	Technique	Reference
porous mesh	polyacrylamide (PAM)	immersed coating	[125]
nano-porous structure	poly(2-hydroxyethyl methacryl-ate-co-ethylene dimethacrylate), poly(butyl methacrylate-co-ethylene dimethacrylate)	in situ polymerization	[143]
nanowire	polypyrrole (PPy)	chemical oxidative polymerization	[144]
carbon nanotubes	poly(d,l-lactide acid, PDLLA)	electrodeposition and immersion	[146]
carbon nanotubes/fibers	poly(butylene adipate-co-terephthalate), multiwalled carbon nanotubes	electrospinning	[147]
hierarchical membrane	*N*-aminoethylpiperazine propane sulfonate (AEPPS) monomer, trimesoyl chloride (TMC) monomer	interfacial polymerization	[140]
hierarchical nanofibrous membrane	polyacrylonitrile (PAN) (pristine NFM)	electrospinning and electrospraying	[141]

**Table 2:** Summary of typical structures and polymers of bio-inspired superhydrophobic structures.

Bio-Inspired Structures	Materials	Surface Modification Technique	Water Contact Angle (°)	Rolling-Off Angle (°)	Reference
polymeric fibers and beads	high-molecular-weight poly(AN-*co*-TMI) and perfluori-nated linear diol (fluorolink-D)	blending and electrospinning	166.7	4.3°	[152]
nanostructure	poly(methyl methacrylate)(PMMA) and polystyrene (PS), perfluorooctyltrichlorosilane (FOTS)	mold transfer	151	Sticky	[153]
micro-/nanostructure	poly(methyl methacrylate) (PMMA), polycarbonate (PC) and cyclo-olefin copolymer (COC), (heptadecafluoro 1,1,2,2-tetrahydrodecyl)trichlorosilane	plasma etching	151	4	[154]
porous multilayers	poly(ethyleneimine) (PEI), poly(vinyl-4,4-dimethylazlac-tone) (PVDMA)	layer by layer assembly	156	1	[155]
porous layer	poly(styrene-*co*-divinylbenzene), poly(styrene-co-divinylbenzene)	polymerization	172		[143]
nanocapsule-coated fabric	polydopamine, octadecylamine	spontaneously deposition	145	Less than 10	[149]
micro-/nano-patterned	polydopamine (PDA)	polymerization and mold transfer	151	180	[156]
porous branched structure	polypropylene, p-xylene	solvent evaporating	160		[157]
nanotube	polystyrene	template	162	180	[48]
nanofibers	poly(vinyl alcohol) (PVA)	template extrusion	171		[49]
Leaf-like microbumps	poly(methyl methacrylate) (PMMA)/silica	uv-radiation	163	4	[158]
porous aerogel	graphene/polyvinylidene fluoride (G/PVDF)	solvothermal reduction	153		[159]
hierarchical fabric films	poly(1,3,5,7-tetravinyl-1,3,5,7-tetramethylcyclotetrasiloxane) (p(V4D4)) layer and poly(1*H*,1*H*,2*H*,2*H*-perfluorodecylacrylate) (p(PFDA)) layer	initiated chemical vapor deposition (iCVD)	154	2	[160]
rambutan-like hollow sphere	polyaniline, perfluorooctane sulfonic acid (PFOSA)	self-assembly	164.5		[161]
hierarchical porous structure	ethylenedioxythiophene (EDOT)	electrodeposition	155		[162]
mesoporous/film	polyvinylidene fluoride (PVDF), mesoporous sub-micron carbon capsules (MCC)	dip-coating	160	5	[163]
bowl-like array structure	polyvinyl alcohol (PVA), silver, 1*H*,1*H*,2*H*,2*H*-perfluorodecanethiol	thermal evaporation, template transfer	163	3	[164]
fibrous texture	polypropylene, polyethylene	laminating exfoliation method	156	5	[165]
nanoscale spherical micelles	fluorinated acrylic copolymer	spray coating	164	1.7	[166]
honeycomb structure	poly(vinyl phenol)-block-polystyrene (PVPh-b-PS)	casting	159		[167]
square-shaped pillar patterns	poly(1-methoxy-4-(*O*-disperse red 1)-2,5-bis(2-methoxyethyl) benzene) (PODR1)	laser microstructuring	157		[168]
nanofibrous protrusions	polypropylene (PP), polyethylene (UHMWPE)	hot press lamination and peeling process	158		[169]
micro-textured	ethyleneglycoldimethacrylate (EGDMA), tertbutyl methacrylate (TBMA), perfluorooctylethyl methacrylate (FMA)	UV light-triggered micro/ nanofabrication	163	1	[170]
hierarchical porous structure	divinylbenzene (DVB) and SiO_2_ composites	hydrothermalndolvent evaporation	161.3	4	[171]
nanoroughness-on- nanopillar hierarchical surfaces	polycarbonate (PC), perfluoropolyether (PFPE), C_4_F_8_	nanoimprinting	170	3	[95]
microporous	polydimethylsiloxane (PDMS) and poly(methyl methacrylate) (PMMA)	spray-coating technique	157.5	2.8	[50]
nanofibers	poly(vinylidene fluoride) (PVDF) membranes	electrospinning	171	1.5	[172]
nanoporous	poly(2-hydroxyethyl methacrylate-co-ethylene dimethacrylate) (HEMA-EDMA) and 1*H*,1*H*,2*H*,2*H*-perfl uorodecanethiol	thiol-yne click-chemistry	170	4.4	[173]
fibrillary structure, cauliflower-like structures	3,4-ethylenedioxythiophene (EDOT)	Staudinger–Vilarrasa reaction and electrodeposition	154.5		[174]
Nano-fibrillary structure	3,4-ethylenedioxythiophene (EDOT)	Huisgen reaction and electrodeposition	159		[175]

**Table 3:** List of bio-inspired structures, materials, surface modification techniques for the fabrication of the bio-mimicked oleophobic materials and the oil(s) contact angles.

Biomemetic Structure	Material	Surface Modification Technique	Oils for Testing	Static Contact Angle (°)	Reference
nanofibers	polymethyl methacrylate (PMMA), fluoro polyhedral oligomeric silsesquioxane (POSS)	electrospin	Hexadecane decane	110 145	[60]
nanoparticle-covered cotton textiles	silica nano particle, cotton textiles, 1*H*,1*H*,2*H*, 2*H*-perfluorodecyltrichlorosilane	dip-coating	sunflower oil hexadecane	140 135	[78]
microfibers	polyester, fluorodecyl polyhedral oligomeric silsesquioxane (POSS)	dip-coating	grapeseed oil	145	[80]
nanoparticles	silica, sacrificial polystyrene, tridecafluoro-1,1,2,2,-tetrahydrooctyl trichlorosilane	UV-ozone treatment and dip-coating	hexadecane	70	[84]
diamond nanograss array	polycrystalline boron-doped film, 1*H*,1*H*,2*H*,2*H*-perfluorodecyltrichlorosilane	dip-coating	hexadecane	100	[81]
inverse-trapezoidal microstructure	polydimethylsiloxane, 1*H*,1*H*,2*H*,2*H*-perfluorodecyltrichlorosilane	plasma treatment vapor deposition	methanol	135	[75]
micropillars roughened with nanoparticels	fluorinated 3,4-ethylenedioxypyrrole	electrodeposition	Hexadecane sunflower oil dodecane	144 153 135	[70]
microbumps	fluorinated poly(3,4-ethylenedioxypyrrole) (PEDOP) derivatives	electrodeposition	hexadecane	157	[75]
mushroom-like micropillar	silicon on an insulator wafer, PDMS, perfluoropolyether, octafluorocyclobutane	vapor deposition	ethanol	150 to 160	[66]
overhang structure	poly (perfluorodecylacrylate)1*H*,1*H*,2*H*,2*H*-perfluorodecyl acrylate	oxygen plasma treatment vapor deposition	mineral oil	110	[207]
